# A Qualitative Study of the Lived Experience of Children with ADHD

**DOI:** 10.3390/bs15121698

**Published:** 2025-12-08

**Authors:** Daphne S. Ling, Adele Diamond

**Affiliations:** Department of Psychiatry, University of British Columbia, Vancouver, BC V6T 2A1, Canada; adele.diamond@ubc.ca

**Keywords:** neurodiversity, neurodevelopmental disorder, strengths-based, creativity, hyper-focus, flow, ADHD, lived experience, self-perception

## Abstract

Attention-Deficit Hyperactivity Disorder (ADHD) is a common neurodevelopmental disorder. While there have been many empirical studies of childhood ADHD, there have been few qualitative studies investigating first-hand accounts of the lived experiences of children with ADHD. This study addresses that gap with qualitative data from open-ended interviews with 12 children ages 8 to 14 years about the positive and negative aspects of their ADHD. Overarching themes were identified using Interpretive Description and Thematic Analysis. An important point to emerge was a discrepancy between the generally negative perception of ADHD by society and the medical profession (a psychiatric disorder that needs to be cured) and the more nuanced perceptions of children who themselves have ADHD, where they express positive as well as negative aspects. Positive aspects reported included having more energy, the ability to hyper-focus, and being more creative and more fun because of their ADHD. The children’s nuanced view of their ADHD is also at odds with an exclusively asset-based neurodiversity perspective that focuses only on strengths. The children perceived that some aspects of their ADHD are not advantageous. Implications are discussed with reference to rethinking how we understand ADHD, treatment practices for ADHD, and how to involve children with ADHD in future research.

## 1. Introduction

Attention-Deficit Hyperactivity Disorder (ADHD) has traditionally been considered a neurodevelopmental disorder characterized by hyperactivity, impulsivity, and/or deficits in components of executive functions (EFs), specifically selective attention, sustained attention, working memory, and/or response inhibition. ADHD often becomes evident relatively early in life and is the one of the most prevalent childhood mental health disorders, affecting about 8% of children globally (Ayano et al., 2023). While ADHD had previously been thought to be a disorder limited to childhood, it is now known that it often persists into adulthood, affecting individuals across the lifespan (review: Faraone et al., 2021). In addition, while ADHD had previously been considered a disorder limited to the West, it is now known that it is found in all ethnicities across the world (Faraone et al., 2021).

In school-age children, ADHD is often associated with poorer academic achievement (Arnold et al., 2020), difficulty with peer relations (Mikami & Normand, 2015), and anxiety and depression (Biederman et al., 1991). Studies have also shown that children with ADHD are more likely to struggle in relationships within their families, such as with their parents (e.g., Lifford et al., 2008) and siblings (e.g., Mikami & Pfiffner, 2008). Individuals with ADHD are also more likely to drop out of school, have lower income, higher job turnover, and higher rates of divorce (Faraone et al., 2021). Studies have shown that there is stigma associated with ADHD, as people with ADHD are often perceived to be lazy or less competent (Godfrey et al., 2021; Speerforck et al., 2019). The intertwining of ADHD with many aspects of functioning, as well as its social impact, are important to understand. Sherman et al. (2006, p. 198), however, raised that children with ADHD also possess strengths as they are “*polyactive, excellent brainstormers, eager to please, energetic, and creative*.” A recent 2023 study by Charabin, Climie, Miller, Jelinkova, and Wilkins (Charabin et al., 2023) looked into this by examining strength and resilience in 56 children (10–17 years of age), 38 of whom had ADHD; the remaining 12 were age-matched controls. The authors found that those with ADHD reported average level of strengths as measured on the Behavioral and Emotional Rating Scale (2nd Edition) Youth Rating Scale, and this was similar to the levels of strength reported by controls. This indicates that children with ADHD see some strengths in themselves that might come from their having ADHD.

One aspect of ADHD that is very much under-studied is first-hand accounts of the experiences of those living with ADHD, especially children. Studies investigating health-related quality of life (QoL) in children with ADHD are more prevalent, but QoL studies employ standardised forced-choice questionnaires rather than open-end questions where children can explain their answers. One commonly used questionnaire is the Pediatric Quality of Life Inventory (PedsQL; Varni et al., 2001), which comes in parent-proxy and child-directed versions. In meta-analyses and systematic reviews, Wanni Arachchige Dona et al. (2023) and Lee et al. (2016) summarized that studies using the PedsQL have generally found that children and adolescents with ADHD report lower QoL, especially in the school and psychosocial domains. There have, however, been reports of discrepancies between QoL as reported by parents and those by the children themselves (Klassen et al., 2006; Lee et al., 2019). This brings up the necessity of not relying too heavily on parental reports and to also incorporate children’s own accounts of their experiences with having ADHD.

We saw a similar trend in studies that employed qualitative methods. Our literature search found that while there are many studies with “ADHD” and “qualitative” in the title, abstract, or body of the text, most of those studies focused on people other than the person with ADHD, such as parents/caregivers, teachers, and/or clinicians. Among studies that have centered on those living with ADHD, most interviewed young adults. Of these, three in particular looked at positive aspects of having ADHD (Nordby et al., 2023; Sedgwick et al., 2018; Schippers et al., 2022). Studies looking specifically at children’s lived experience with ADHD, however, are few and far between. Even more scarce are any qualitative studies that investigated positive ways that children with ADHD view themselves. Indeed, a meta-synthesis of studies of the experiences of children (defined as 19 years and younger) with ADHD published in English turned up only 16 studies, four of which were doctoral dissertations (Ringer, 2020). Of the 16 studies in Ringer’s meta-synthesis, *only two* (the doctoral dissertation of Grant, 2009, which looked at 8 youths between the ages of 11 and 18, and the Walker-Noack et al. (2013) study that looked at 25 youths ages 10 to 21) identified themes of *positive* experiences of children with ADHD. The dissertation by Grant was not available to view, request, or order and the author only had the dissertation indexed in the library and could not be traced. Across the 16 studies, children and youth spoke of their concept of self (e.g., J. Kendall et al., 2003; Knipp, 2006; Krueger & Kendall, 2001; Singh et al., 2010), difficulties in controlling their behavior and attention (e.g., Gallichan & Curle, 2008; J. Kendall et al., 2003; L. Kendall, 2016; Leyland, 2016), as well as needing to adapt to the environment. The children also spoke of living with stigma (e.g., Honkasilta et al., 2016), struggling to fit in (e.g., Gallichan & Curle, 2008; Grant, 2009; Hallberg et al., 2010), difficulty living up to expectations, rejection, and isolation (e.g., Levanon-Erez et al., 2017; Wiener & Daniels, 2016), and how others in their environment helped them (e.g., Grant, 2009; Walker-Noack et al., 2013).

We also looked a little deeper to find studies that examined first-hand perspectives of children with ADHD using formats other than the more traditional interview. We found one paper (Barfield & Driessnack, 2018) that reported a draw-and-tell study. The authors reported that children with ADHD found happiness in their relationships with others. There was also a study where researchers very cleverly gave 16 children with developmental disabilities (8 of these children had ADHD or ADHD with comorbid disabilities) a digital camera for them to take photos and videos of their lives in three different contexts: home, school, and community (Coussens et al., 2020). These photos and videos were then followed up with interviews. The authors were interested in seeing how children themselves viewed their ability to participate in activities and their environment. They found that children were happy with their participation when they were able to play with their friends, learn a new skill (especially in sports or at school), and be included in family events. We also found an illustrated report by Singh (2012) through ADHD VOICES, based in the UK, which examined childrens’ perspectives on self-authenticity, moral agency, personal responsibility, and societal issues from an ethical standpoint. Children in the study were from the US (n = 69) and the UK (n = 82) in this report. While there were regional differences, the children as a whole reported experiences of stigma, how medication affected their behaviour and their sense of self, as well as their understanding of what constitutes good and bad behaviour.

Several different methodological frameworks have been used by researchers conducting interviews on ADHD. These include Interpretive Description (e.g., Lane-Barmapov, 2016), Interpretive Phenomenological Analysis (e.g., Redshaw & McCormack, 2022), and Phenomenology (e.g., Sedgwick et al., 2018). Of these frameworks, the one that is most relevant to this study is Interpretive Description (Thorne, 2017). Interpretive Description acknowledges that patients are experts on their own lived experience and that how they view those experiences provides valuable insights (Thorne, 2017). Interpretive Description’s goal is to inform and shape clinical care. Because its aim is shedding light on lived experiences, Interpretive Description lets the patient lead the way and is not tied to a priori hypotheses or theories. It is also an appropriate approach here because a small sample size (such as in the present study) is not an important or limiting factor for this method (Burdine et al., 2020).

We could find no Interpretive Description studies that have been conducted with children with ADHD, nor of persons of any age with ADHD. The closest we could find were two studies on a neurodevelopmental disorder (autism), which is often studied alongside ADHD and can co-occur with ADHD. The two studies were of (a) Montessori teachers teaching children on the autism spectrum (Lane-Barmapov (2016), who interviewed 7 children) and (b) parents’ experiences parenting children with autism (York (2007), who interviewed 13 parents).

To address the gap in the literature on aspects of ADHD that children and youth like, are proud of, or find to be positive, we specifically asked children and youth in our study about those things in addition to asking about problems they have, their stressors, and what they struggle with.

## 2. Methods

### 2.1. Study Participants

All 12 children (3 girls, 9 boys) in this study (mean age = 11.6 years) had been previously diagnosed with ADHD as per the guidelines of DSM-5 (DSM = Diagnostic and Statistical Manual of Mental Disorders) by a children psychiatrist and were currently taking at least 20 mg of either Methylphenidate (Biphentin^®^) or Lisdexamfetamine Dimesylate (Vyvanse^®^). They were also under the care of a physician who had no objections to their participation in this study. The twelve children ranged from 8 to 14 years: One child was age 8, two age 9½, two age 10, while most (seven) were between the ages of 12 and 14. Thus, the majority of children were in middle childhood to early adolescence and thus capable of self-evaluation.

All children were part of a mixed-methods, crossover-design study looking at the effects of a full dose of psychostimulants on the executive-function performance of children medicated for ADHD (full dose being defined as the current dose the child has been prescribed and which the child is currently taking and has been stabilized on) versus half of that dose. Neither the children, their parents, nor any of the research team were privy to information on which session a child was given that child’s usual dose or half the usual dose. The quantitative results of the study are being reported separately. All children could understand and converse in English, had normal or normal-with-correction hearing and vision, and by parental report had an IQ > 90. None had suffered a concussion or lost consciousness from a fall or blunt trauma to the head.

### 2.2. Study Procedures

This study was approved by the University of British Columbia’s Clinical Research Ethics Board (H14-00224), British Columbia Children’s and Women’s Hospital Research Ethics Board (CW14-0118), and Vancouver Coastal Health Research Institute (V14-00224).

We used Interpretive Description (Thorne, 2017) and Thematic Analysis (Vaismoradi et al., 2013) to guide the interviews and analyses in this study.

As we were interested in hearing from the children about their lives and their experiences living with ADHD, we administered six very short open-ended age-appropriate interview questions about their ADHD. No follow-up probing was done in the interviews. This interview was administered at the beginning of the testing day after consent and assent had been obtained. To move away from a deficit-based approach to a strengths-based approach, we included three questions in the interview that allowed children to tell us about themselves independent of their ADHD. The qualitative questions on their experiences with having ADHD were as follows: (1) *My ADHD symptoms are __(answer)__*. (2) *Are there aspects of ADHD that you like? If so, what are they?* (3) *What aspects of ADHD bother you most?* (4) *What things do you most like to do?* (5) *What are you most proud of?* (6) *What things have caused you stress or worry over the past 12 months, including currently?* Questions 1–3 related to the children’s experience of their ADHD specifically. Questions 4–5 related to positive things in their lives generally. Question 6 related to stresses and anxieties that trouble them. The interviews were conducted by the first author (DSL) and a research assistant, Kristina Balce. They were typed up by DSL. Both DSL and KB were supervised by the senior author, AD.

### 2.3. Data Analysis

To prevent being steered in a given direction prematurely, we waited until the end of the study before reading and typing up the interviews with participants. The verbatim responses from each child appear in the Supplementary Materials. Once the responses were typed up, DSL read through them to develop a preliminary sense of what the children were conveying. After that, DSL started to code for similarities across answers to identify recurring patterns and themes throughout the answers, a process called thematicanalysis (Vaismoradi et al., 2013). This is elaborated further below.

One variable to be aware of in the interviews is the researcher’s positionality in the interviews, as we all carry with us inherent biases from our personal experiences (observer bias; Mahtani et al., 2018) and the observer or researcher always influences what phenomenon is observed. Terms for these effects include the Heisenberg Principle (that the act of observing something influences what is being observed; Dirac, 1967) and the Hawthorne effect (study participants behaving differently because they know they are being watched; McCarney et al., 2007). Interpretive Description methodologies acknowledge that the researchers’ experiences and personal characteristics and biases play an important role in informing research as pre-existing knowledge in a field informs understanding of the factors that affect participants’ perceptions of and feeling about their experiences (Burdine et al., 2020) and perceived attitudes and beliefs of the researcher affect what participants choose to reveal (Bergen & Labonté, 2020). To prevent DSL’s biases from unintentionally skewing interpretation of the data too much, the transcripts were passed on to another researcher who was not familiar with the study and had had no contact with the participants, for independent interpretation and coding of the data. DSL’s coding and interpretations were not shared with this other person. Once the person had written down her general impressions, she then grouped the answers for each question into general themes before handing them back to DSL. General themes were generated based on answers that had considerable and broad overlap. After receiving her list, DSL then went back to the themes she had originally generated and compared the two. Overall, there was excellent agreement between the coding of the other researcher and DSL, although there were minor instances where they generated different groupings. One example is when DSL grouped pride in playing sports and feeling skilled at playing sports together, but the other researcher grouped feeling skilled at playing sports (e.g., “*Good hits at baseball*”) and pride in playing sports (e.g., “*I am proud that I play Division 1 soccer*”) separately. DSL then narrowed down the themes by collapsing those that were similar. Finally, DSL looked at all the answers to all questions as a whole and generated overarching themes and went over those with AD (her supervisor), who helped to narrow down and characterize the themes further.

## 3. Results

Qualitative data from the 12 children (3 girls) are presented here. The children were between the ages of 8 and 14 (mean age: 11.5 years), and seven of them were interviewed by DSL and five by KB. Seven of the 12 children (58.3%) were of European descent. The remaining six children were of East Asian (n = 2), South Asian (n = 1); Latin (n = 1), European-East Asian (n = 1) descent; one child was adopted and her parents did not know her roots. A majority of the parents (83%) had at least a college-level education. Ten of the 12 children lived in two-parent households. Of the remaining two, one child split his time between his father and mother (both of whom have remarried) and the other child lived full-time with his mother. Six of the 12 children were from upper-middle/upper class (household income of >$100,000) while the remaining were middle class (n = 3; household income of $50,000–$100,000), lower-middle class (n = 2; household income of $20,000–$49,999), and one (single) parent was unemployed.

Seven children chose to dictate their answers to the researcher, three chose to write their answers down themselves, and the other two children chose to do a combination of each. There was a possibility that children who chose to write their answers down might provide less information than children who chose to dictate to the researcher as children with ADHD have been known to struggle with writing and expressing themselves (e.g., Molitor et al., 2016). However, given that the children who chose to dictate their answers did not give longer responses than those who chose to write, this was probably not a problem in this study.

We will follow the convention throughout of referring to each participant by a letter from A to L, prefaced by S for “subject.” Only one child (SD) had a diagnosis of anxiety. None had a diagnosis of depression. Five parents, however, reported their child had a tendency to be anxious and two of those five parents also mentioned their child had a tendency to be depressed. Two of the children (SB and SD) were reported to have Oppositional Defiant Disorder. SD’s parent commented that SD’s defiance seemed to be tied to his anxiety as it manifested when SD was feeling anxious and having trouble regulating his emotions.

Although the method used to arrive at the themes was in line with Interpretive Description, we have chosen to follow the more standard practice in scientific papers of leaving discussion of the results for the Discussion section, versus the norm in Interpretive Description studies of interweaving discussion with results in the Results section.

With the exception of one child (SG, who was diagnosed as having ADHD of the inattentive type), all the children had combined-type ADHD. Seven children reported symptoms of hyperactivity and seven reported attention-related symptoms (five reported attention impairment; two reported they had superior attention). Only four children shared that their symptoms included both hyperactivity and attention-related symptoms. Interestingly, one of those four children was SG who was diagnosed as have the primarily inattentive type of ADHD. An example of what children shared when they mentioned both types of symptoms is this provided by SL, who said, “*Not being able to focus. Being impulsive and not being able to sit still*.” Another child (SC) echoed similar issues, “*Get distracted, mostly at school, but everywhere too. Too much energy; can’t sit still*.”

Several themes were identified across all six questions.

### 3.1. THEME 1: ADHD as Both an Advantage and a Disadvantage

A prominent theme was the ambivalent attitude and feelings children had toward their experience of ADHD. They described perceiving their experience as both positive and negative, even with regard to the same topic.

#### 3.1.1. Subtheme 1a: The Two Faces of Attention in ADHD

Five children mentioned that their symptoms included impairments in attentional control, but only two of those five children (40%) mentioned that it bothered them. Seven children mentioned attention-related issues as symptoms of their ADHD, yet two of those children (29%) also mentioned that being able to hyper-focus was something they liked about their ADHD. They saw it as one of their strengths. For instance, SK wrote, “*That I can consentrat [sic] better*.” When asked about his symptoms, adding “*that it makes* [me] *creative, daydreaming, consentrating [sic]*.” Another child (SL) mentioned a similar combination when asked about his ADHD symptoms. He reported impaired attention as one of his symptoms (“*Not being able to focus*”), but noted that he liked having especially strong concentration skills (“*My hyperfocus*”) when asked what he liked about having ADHD. SG also expressed having two sides to his attention (hard time focusing, but then staying focused on some random thing, not necessarily what he wanted to be focused on) saying: “*Very hard to focus when I’m working; usually I’m thinking of one thing and then I can’t stop thinking about, usually random things*.”

#### 3.1.2. Subtheme 1b: The Two Sides to Hyperactivity in ADHD

Of the 12 children in this study, seven (58%) mentioned hyperactivity and increased energy as a symptom. Some of what children liked about their ADHD, e.g., having a lot of energy, seemed to be related to their hyperactivity. For example, when asked what he liked about his ADHD, SI said “*lots of energy*” and went on to add “*I don’t know if it’s ADHD but I never want to go to sleep.*”^1^ Five of these seven children (71%) also said they liked having the extra energy for various reasons. For example, when asked what he liked about his ADHD, SG said, “*It makes me energetic and makes me better at baseball and sports and I can cheer my teammates on at every single pitch and it’s because of my ADHD.*” Another child (SD) shared the two different sides of hyperactivity in his answers. When asked what his ADHD symptoms were, he responded “*Don’t really know; without meds, get crazy and annoying*.” However, when SD was then asked if there were aspects of ADHD that he liked, he said, “*More fun and active, sometimes taking meds make me boring*.” Indeed, three children (25%) said they liked being creative, two of whom mentioned hyperactivity in the same answer. One child (SE), for instance, talked about her creativity, sharing, “*I like how I think outside the box a lot…I come up with answers that others don’t*. *It makes me more hyper than other kids my age.”* SF shared similar feelings when she said, “*When hipper* (sic; hyper)*, creative, flexible, athletic.*” As mentioned above, the third child included here (SK), said that he liked “*that it makes me creative, day dreaming, consentrating* [sic],” but did not specifically mention hyperactivity in any of his answers.

#### 3.1.3. Subtheme 1c: The Two Sides to Relationships in ADHD (Causing Both Stress and Joy)

Relationships were very important to many of the children; they thrived when able to have positive relationships with others. A downside of ADHD for many was that their ADHD negatively affected how they interacted with others causing problems with relationships. Thus, relationships were a source of both joy and stress for many of the children in our study.

With the exception of schoolwork (which 7 children [58%] mentioned as a source of stress; see Theme 3 below), stressors for the children all revolved around social relations: relationships at home (with parents for four of the children, with siblings for two of the children, and others at home for two of the children) and/or within friends (mentioned by two children). The relationship stressors broadly formed two categories (how events happening with others affected the child and the child’s concern about getting along with others). One example of the former is when one child (SF) shared, “*when my mum and dad fight*” in response to what causes him stress or anxiety. A second child (SG) shared, “G*randpa was sick and has gone to the hospital 5 times and we didn’t know if he would survive but somehow he did and he might go back again. Grandma died in 2014. I was thinking of it.*” In the latter category, one child (SI) said, “*People excluding me from games*.” Another child (SD) said, “*arguments with friends at school*.” The same child later shared, “*Sometimes friends don’t like hanging out* [with me] *because* [of my] *ADHD*”.

When asked what aspects of ADHD bothered them, children mentioned both things that affected them and ways in which they perceive they annoy or bother others. For example, as mentioned above, SE said, “*The lack of patience, can’t control my emotions. If my brothers are being super annoying, it’s harder to walk away*.” Again, as mentioned above, SL expressed that his inability to stay still was annoying to others, “*Not sitting still. It is annoying to me as well as others*.”

Although children expressed experiencing stress in connection with their relationships with others, it was also clear that their relationships with others brought them joy. For example, SJ shared, “*I like to play soccer and video games. I also like hanging out with my friends.*” Another child (SC) said, “*Swim, snowboard, be with friends, play video games and being active.*”

### 3.2. THEME 2: Activities That Give the Children Joy and Pride

It was apparent from the children’s responses that sports, play, and music were important to them. These brought both joy and pride to many. More than half the children expressed joy in physical activity (n = 8, 67%), such as sports and/or other forms of physical activity (e.g., climbing trees). All 12 of the children in the study played at least two sports (range: 2–6 sports), so it is not surprising that sports emerged as something they commonly mentioned enjoying. Eleven of the 12 children also mentioned they liked attention-relieving activities such as video games and watching TV (n = 9; 75%) and other forms of indoor play such as Legos (n = 5; 42%), with two of the 11 children mentioning both.

All of the children in the study, with the exception of one (SH, who mentioned only video games), shared more than one thing that they enjoyed. An example of a child who mentioned finding joy in both more physically active and more attention-relieving activities was SL, who said, “*Swim. Climb trees. Play video games and listen to music, also biking*.”

Half the children in the study (n = 6) played at least one instrument (range: 1–3 instruments), and one took voice lessons (no instrument). Half the children mentioned that being able to play sports and/or music was a source of pride for them. For example, SF cited “*my soccer skills*” when asked what she was proud of, and also mentioned liking multiple types of activities (“*Soccer, watch YouTube, gymnastics*”). Another child (SJ) shared, “*I am proud that I play Division 1 soccer*.” One child (SG), in response to being asked what he was proud of, said, “*Playing the drum. I’ve been playing it for 4 years; played it in front of my whole school 4 times.*”

### 3.3. THEME 3: Schoolwork and Homework Were Stressful

Seven children (58%) mentioned that schoolwork was a source of stress, especially homework, with one child mentioning teachers as a source of stress. That only one child mentioned teachers as a source of stress speaks well of their teachers. SA, who mentioned both schoolwork and the teacher shared, “*Homework, teachers, & everything else. The books make no sense, too much, too difficult, the teacher doesn’t understand me. The teacher talks too much.*” Out of the other six children who mentioned school-related work as a source of stress, three mentioned “*homework,*” one mentioned “*math,*” and two mentioned “*school*.”

### 3.4. THEME 4: Difficulty with Articulating Their Experiences

The last theme to emerge was that children appeared to have difficulty articulating what their ADHD symptoms were or what about themselves they would attribute to their ADHD. These difficulties in articulating themselves were specific to ADHD because none of the children had trouble when asked questions about what brought them joy or pride or stressed them out. One child (SH) responded with “*I don’t know*” to questions about his ADHD symptoms and what he liked or disliked about his ADHD. A second child (SA) said “*not sure*” (concerning his ADHD symptoms), “*don’t really know*” (about what they liked about ADHD), and “*no*” (about what bothered him about his ADHD).^2^ Another child (SK) left the question about what bothered him about his ADHD blank.

While 9 of the 12 children (75%) could find something they liked about their ADHD, three children (SB, SC, and SJ) could not come up with anything they liked about their ADHD, responding, respectively, “*not that I can think of,*” “*nothing in particular,*” and “*not really.*” On the other hand, one child (SC) indicated that there was not anything about his ADHD that bothered him, replying simply “none” to the question about what aspects of ADHD bothered him, another child (SA) replied “no” when asked what bothered him about having ADHD, and yet another child (SK) replied to that question by not writing anything (leaving his answer blank).

## 4. Discussion

This study is based on the perceptions and lived experiences of children with ADHD regarding their symptoms, positive and negative aspects of their having ADHD, and positive and negative aspects of their lives (such as things that brought them joy, pride, or stress). Short open-ended interview questions were administered to 12 children with ADHD and the transcripts were analyzed in an inductive manner to extract themes and commonalities and differences among the children’s responses. These analyses were conducted independently by two separate researchers, with strong agreement between them. Not all the themes uncovered are specific to ADHD. For example, many children without ADHD also enjoy playing sports and find school stressful.

Overall, this study raises several important key points. One important point that emerged is that there appears to be a discrepancy between how society and the medical profession seem to perceive ADHD, which is as something negative (a disorder, “a psychiatric condition” that needs to be “cured”), and the more nuanced perceptions of children who themselves have ADHD, where they express not only negative aspects of it, but also positive aspects.

For example, children in our study did not necessarily perceive their hyperactivity as something negative. Similarly, Nordby et al. (2023) and Schippers et al. (2022) found that adults with ADHD enjoyed having hyperactivity as it helped with activities like sports and trying new things.

Several of the children in our study mentioned that they felt they had more energy because of their ADHD and shared that they enjoyed having this energy. Walker-Noack and team (2013) found that, although the 25 youths 10 to 21 years whom they interviewed seemed to have difficulty coming up with positive aspects of having ADHD (with three specifically said there were no positives), 72% of them shared that their ADHD made them more energetic in response to the question about positives. This contrasts with the normally negative connotation that “hyperactivity” carries, as behavior that is excessive, “over the top,” out of control, and/or inappropriate. This is not to say that the children who we interviewed did not also perceive negative aspects to their hyperactivity, but they also mentioned that it helped them with sports, for example. And, many of the children also shared that sports and play brought them joy and pride.

McClure (2013), a child and adolescent psychiatrist, has noted that ADHD is rather unique among child and adolescent psychiatric conditions because the “impairments” are seldom raised as complaints by the children and adolescents themselves. Rather, the issue is brought to the attention of the clinician through reports by parents and teachers, which are then used as diagnostic criteria. Our data would suggest that there is at least some truth in this critique as what was reported as a problem by adults (about the child) did not necessarily translate to what the child felt and experienced. This was raised by one child (SB) who shared that “*It doesn’t* [bother] *me, but* [it] *bothers my teachers. I hear about it in my report card and I feel bad about it.*”^3^ Another example was when child SA said “*Not sure*” in response to what her symptoms were. SA’s mother was in the room, and it was she who responded with “*Hard time staying focused, fidgets, defiant, hard time concentrating, hard time listening*.” In analyzing the children’s answers in this study, we wondered how much of what the children expressed about their ADHD symptoms was a product of being told what ADHD is, as opposed to what they actually experience. That might be a fruitful area for further study.

Another plus that children attributed to their ADHD was their ability to hyper-focus (to focus with particular intensity, screening out all distractions). At least two studies with adults with ADHD also found that being able to hyper-focus was seen as a positive aspect of their ADHD (Nordby et al., 2023; Schippers et al., 2022). When one hyper-focuses, one is so completely absorbed with what one is doing that one completely screens out everything else. While hyper-focusing has often been described as a negative in the research and clinical literature as an unhealthy over-focusing (e.g., oblivious to things in their environment, so focused one cannot get their attention when needed or so focused that they even ignore bodily signals, like needing to go to the bathroom) one can also see this as closely akin to something that has universally been considered a positive, i.e., being in a state of “flow” (Csikszentmihalyi et al., 2005). Flow is being so completely immersed in, and focused on, what you are doing that you may not notice the passage of time or anything in your surroundings. Flow is broken when you step outside the activity to evaluate how you are performing or notice anything about your body or emotions. It is being so at one with the activity you are doing that you are oblivious to everything else. The positive aspects of hyper-focusing, and its similarities and differences with flow, seem worthy of further study.

Relationships with family, friends, and in some cases, the community at large, seemed to be a double-edged sword for the children in our study too, as social relationships brought them joy but was also a source of stress. This was seen in several children who mentioned doing activities with people in their social circle when asked what they enjoyed doing and/or what they were proud of, and later also referenced people in their social circles when asked about their sources of stress. Our findings here partly echo the finding of Barfield and Driessnack (2018); mentioned in the introduction) who found that children noted that their relationships with others made them happy. Unlike Barfield and Driessnack, however, we also found that those relationships can cause the children stress. Barfield and Driessnack only asked children about positive aspects of their ADHD, whereas we asked about both positives and negatives.

Several children interviewed for this study perceived themselves as more creative and more fun *because of* their ADHD, which also echoes findings in adults (Nordby et al., 2023; Schippers et al., 2022) and in youths (Walker-Noack et al., 2013). Although we did not assess creativity in this study, evidence from other studies suggests that psychostimulant medication does interfere with creativity in children with ADHD. González-Carpio Hernández and Serrano Selva (2016) found that in children with ADHD aged 8 to 12 years, psychostimulant medication (Methylphenidate) resulted in lower scores on several domains of creativity as measured by the Torrance Figural Tests of Creative Thinking (TTCT). The domains affected were in Creative Strengths, Fluency, Originality, and the overall (Global) Creative Index. Ten et al. (2020) similarly found that children with ADHD (also 8–12 years of age) who take psychostimulant medication scored lower on measures of both open-ended and close-ended creativity compared to children who do not take medication. This seems to be worth looking into further.

Given (a) that children with ADHD in this study enjoy the extra energy, not being boring/being more fun, being more creative, and being able to hyper-focus, and (b) that those traits have been found to contribute to persons with ADHD doing well (Bergen & Labonté, 2020), it might be worthwhile to consider prescribing lower doses of psychostimulants to help retain the aspects of ADHD that those with ADHD enjoy and find to be beneficial. And, it might be worthwhile to complement prescribing medications to mitigate ADHD symptoms with more attention to environmental accommodations to enable children with ADHD to shine, which in some cases can obviate the need for medication altogether. Perhaps we can re-think learning environments so they not only accommodate differences but embrace talents, as the neurodiversity movement has advocated. A simple environmental accommodation would be allowing children who are hyperactive to walk around more during lessons, and allowing all children more time for exercise and recess, which has been shown to be beneficial for the academic performance of most students (Pellegrini & Bohn-Gettler, 2013). Also, corporations could leverage the talents of those with ADHD and others who are not neurotypical by altering their environments so they are more flexible, allowing for different styles and approaches. Such initiatives could spur economic growth by attracting diverse talents to solve real-world problems.

Pointing out positive aspects of their ADHD, as the children in this study did, is consistent with a neurodiversity perspective, which argues for rethinking the way we understand conditions such as ADHD and autism, seeing these as differences rather than as deficits, and as has having unique gifts and strengths (Botha et al., 2024; Lai et al., 2025; Sonuga-Barke & Thapar, 2021). It asks: Might the achievements of neurodiverse individuals take place, not despite their conditions, but *because of* them? Singer (2016, p. 87), an early adopter of the term “neurodiversity,” wrote, “*Why not propose that just as biodiversity is essential to ecosystem stability, so neurodiversity may be essential for cultural stability?*” This perspective helps individuals with ADHD, or any other type of neurodiversity, to see themselves through a strengths-based or assets-based perspective, enabling them to see themselves as capable rather than as problems to be solved. Both hyper-focusing, on the one hand, and distractibility and an inability to sustain attention, on the other hand, are characteristics of attention that are often true of children with ADHD. The children can be easily distracted and have difficulty staying focused on something they are not super-interested in, but for something of genuine interest they can be remarkably focused for remarkably long periods of time (Ashinoff & Abu-Akel, 2021).

The more nuanced view of their ADHD expressed by most children interviewed for this study (perceiving pluses as well as minuses to their having ADHD) is at odds with a solely assets-based neurodiversity perspective (e.g., Lai et al., 2025) that focuses only on strengths. The children interviewed here perceived that some aspects of their ADHD are not good and can be disadvantageous.

We would like to draw particular attention, however, to our findings concerning what the children perceived as the positive aspects of ADHD, such as having more energy, being able to screen our distraction particularly well, and being especially creative because when a diagnosis is framed solely in terms of deficits or what a person cannot do or is not good at, it can lower a person’s self-esteem and the expectations the person has for him– or herself. The expectations people have for themselves often become self-fulfilling prophecies (e.g., Good et al., 2008). The expectations people have for themselves, especially young people, are crucially shaped by the expectations that others have for them (Cooley, 1902; Rosenthal & Jacobsen, 1968). If others expect less, they may achieve less. Conversely, if we acknowledge their strengths and believe in their potential, they are far more likely to excel. Reducing the stigma, stereotypes, and discrimination surrounding ADHD among educators, health professionals, and the general public (Sonuga-Barke & Thapar, 2021) could go a long way to helping those with ADHD reach their full potential. As Goethe (1795) said, “If we treat people as if they were what they ought to be, we help them become what they are capable of becoming.”

## 5. Limitations

There were several limitations to this study. One was the small sample size. Even though a small sample size is not in itself a limitation in Interpretive Description, we caution readers that it does mean that the results cannot be generalized to the population. As mentioned above, this report is part of a double-blind crossover study examining the effects of the full (currently) prescribed dose of psychostimulant versus half that dose on the EFs of children with ADHD. The small sample size we due in large part to parents’ reluctance to take their child off of their ADHD medication for even two days (the ‘wash-out’ period required before each of our two testing sessions) and parents’ reluctance to tamper with the dose their child was taking even for a single day (i.e., halving it). In looking at previous qualitative studies in children and youth with ADHD, however, a small sample size is a common theme. Ringer’s (2020) meta-synthesis of 16 studies, 14 of those studies had a sample size of 16 or fewer. The smallest sample size was a mere four children, and the most common sample size was 12.

Second, the qualitative questions we posed to the children were very brief and included no follow-up probing, as this was not the main focus of the study and the EF testing was already quite long. In retrospect, there are certainly other questions we would like to have asked. Future research could include asking children about their experiences with their medications, what they felt were positive and negative outcomes of being on medication, and factors they felt alleviated their stress. We should have also probed what children meant by some of their answers. We regret that we did not ask any follow-up or clarification questions and that we conducted only one interview session. One example when follow-up could have provided needed clarification is when child SB said “*focus*” (just the one word alone) when asked to describe his ADHD symptoms. His mother, who was in the room (as the participants in the study are minors, they have the choice to have their parent in the room), immediately said “*Easily distracted.*” DSL interpreted the word “focus” as a positive comment (i.e., a good ability to focus), which would contradict his mother’s response. AD, on the other hand, interpreted it as the child using the one word to indicate that one of his ADHD symptoms was difficulty with focusing (as he never mentioned anything positive about his ability to focus throughout the brief interview), which would match what his mother said. Without follow-up, it is not possible to tell which interpretation is correct, or if there was some other explanation for his answer. Another example of the many instances when follow-up could have been helpful is when subject (SK) left his answer blank when asked about what bothered him about his ADHD. Did leaving the answer blank mean that nothing bothered him about his ADHD, that he had difficulty articulating what bothered him about his ADHD, or something else? We cannot know. Hence, we mentioned this response in Section 3 both under difficulty articulating feelings about their ADHD and under overall feelings about their ADHD.

We also acknowledge that while our intention was to keep the questions open-ended to not push the children in a certain direction, the questions did contain words that indicated we were looking for both positive and negative experiences. This raises the possibility that children who might not have considered a positive or negative aspect to their ADHD now were encouraged to consider that in order to answer our questions. We felt, however, that asking only completely neutral and open-ended questions in the interview with no follow-up, such as the first question in our study (“My ADHD symptoms are ___”), would have elicited blank responses

## 6. Future Directions

Collaborative inquiry or co-production might be a useful approach in further understanding the lived experiences of persons of all ages with ADHD. Collaborative inquiry has been used primarily in educational research studying educators, where the researcher works alongside the study participants. Instead of doing research on teachers to produce knowledge about them, the researcher strives to form partnerships with the teachers to together better understand their interests and needs. The research process is systematic, iterative, and collective. The researcher does not take for granted his or her understandings, and is attentive to the risk of over-interpreting, assuming too much, or speaking for the educators in the study (Elliott, 1985; Pedretti, 1996). This is approach can similarly provide novel insights in the field of mental health (Braun & Clarke, 2019).

During the design phase of our study, we consulted researchers, child and adolescent psychiatrists, psychologists, and pharmacists. We did not consult children with ADHD, however, about how their ADHD and the pharmacological treatment they were receiving affected them, and what it is that they thought was important for us to research. Involving persons with ADHD at any age from the start is one of the things that might be a very useful approach in research going forward. One might start, for example, by inviting children with ADHD to speak with the researcher(s) about their thoughts and ideas (i.e., child-friendly focus groups). An important feature of such research would be funds to compensate families properly, instead of token honorariums, in line with a key principle of this type of research, which is respecting their time and knowledge. Note also that having a parent in the room probably influenced the children’s responses and future research trying to get out the children’s own perceptions and feelings might consider asking parents, provided the child is comfortable with this, to wait outside.

Lignou et al. (2019) and others have similarly advocated for the active involvement of individuals from the clinical population being studied as co-researchers. This co-production approach involves the researcher and individuals living with the condition actively collaborating in the research. This approach starts from the premise that both parties are equal and bring different skills and expertise. The researcher contributes expertise in research methods, safety, and ethical oversight, while the persons with the clinical condition under study contribute their lived experiences. Together they work on developing research questions and methodology. This corroborates with Interpretive Description, recognizing that patients are experts on their own lives. Co-production takes this further by working together with patients from the start in all aspects of the work, as in collaborative inquiry with educators. If we could do this study all over again, this is a route we would have taken.

## Data Availability

The original contributions presented in this study are included in the article/supplementary material. Further inquiries can be directed to the corresponding author.

## Notes

1 Difficulty falling asleep is a common problem in ADHD, often seen as a side-effect of medication, and not necessarily related to hyperactivity.

2 As noted below, this might indicate that nothing bothered the child about his ADHD rather than a difficulty articulating what bothered him.

3 This child was the only one who mentioned this contrast specifically.

## Supplementary Materials

The following supporting information can be downloaded at: https://www.mdpi.com/article/10.3390/bs15121698/s1. Transcripts of the interviews are in the Supplementary File.
