# Supplementary material for: A Qualitative Study of the Lived Experience of Children with ADHD
Source: Behav Sci (Basel). 2025 Dec 8;15(12):1698. doi: 10.3390/bs15121698 (PMC12729330; doi:10.3390/bs15121698)
Supplement: Supplementary file 1 [file behavsci-15-01698-s001.zip › behavsci-3973248-supplementary.pdf]

A Qualitative Study of Lived Experience in Children with ADHD

Supplementary Material

Verbatim Responses and Experimenter Notes from each of the Twelve Interviews

<u>Referred to as SA in paper (Tested by DSL; Answers dictated to DSL)</u>

My ADHD symptoms are:
Not sure
(Mother chimed in: Hard time staying focused, fidgets, defiant, hard time concentrating, hard time listening)

Are there aspects of ADHD that you like? If so, what are they?
Don't really know

What aspects of ADHD bother you the most?
No

Do you play a musical instrument? <u> Yes</u> No If Yes, which
Clarinet

Do you play any sports? <u> Yes</u> No If Yes, which
Gymnastics, swimming, wants to start horseback riding

What things do you most like to do?
Play Lego, go on the trampoline, play dolls, annoy my little sister

What things have caused you stress or worry over the past 12 months, including currently?
Homework, teachers, & everything else. The books make no sense, too much, too difficult, the teacher doesn't understand me. The teacher talks too much; 150 words/min based on dots.
DL asked: Dots?
Child: I make dots of how many words she said.

What are you most proud of?
Proud that my fish died. It jumped out of its bowl.

Experimenter notes

Age and gender	10; Girl
Sports	Yes (2)
Music	Yes (1)
Parent income	$50-100K
Languages	2 (English, French)

Parent education	2-Year College - Degree (mother)<br>High School (father)
Ethnicity	European (mother); East Asian (father)

Referred to as SB in paper (Tested by DSL; Answers dictated to DSL)

My ADHD symptoms are:
Focus
(Mother chimed in: Easily distracted)

Are there aspects of ADHD that you like? If so, what are they?
Not that I can think of

What aspects of ADHD bother you the most?
It doesn't bother me but it bothers my teachers. I hear about it in my report card and I feel bad about it
(Mother chimed in: He gets reminders and is pulled aside)

Do you play a musical instrument? □ Yes □ No If Yes, which

Do you play any sports? □ Yes □ No If Yes, which
Swim, Hockey

What things do you most like to do?
I like to play sports and play video games with my friends

What things have caused you stress or worry over the past 12 months, including currently?
Some things that have caused me stress over the past 12 months are math tests…sometimes my mum.

What are you most proud of?
(No answer)

Experimenter notes

Age and gender	13; Boy
Sports	Yes (2)
Music	No
Parent income	None given
Languages	1 (English)
Parent education	High-School Diploma (mother; single parent)
Ethnicity	East Asian (mother); no information for father

Referred to as SC in paper (Tested by KB; Answers dictated to KB)

My ADHD symptoms are:
Get distracted, mostly at school, but everywhere too. Too much energy; can't sit still

Are there aspects of ADHD that you like? If so, what are they?
Nothing in particular
What aspects of ADHD bother you the most?
None

Do you play a musical instrument? □ Yes □ No

Do you play any sports? □ Yes □ No
Soccer and baseball

What things do you most like to do?
Swim, snowboard, be with friends, play video games and being active

What things have caused you stress or worry over the past 12 months, including currently?
My parents

What are you most proud of?
Me being focused

Experimenter notes

Age and gender	13; Boy
Sports	Yes (4)
Music	No
Parent income	$20-50K
Languages	2 (English, Hungarian)
Parent education	2-Year College - Degree (mother)<br>2-Year College-No Degree (father)
Ethnicity	Eastern European (mother); Western European (father)

Referred to as SD in paper (Tested by KB; Answers dictated to KB)

My ADHD symptoms are:
Don't really know; without meds, get crazy and annoying

Are there aspects of ADHD that you like? If so, what are they?
More fun and active, sometimes taking meds make me boring

What aspects of ADHD bother you the most?
Sometimes friends don't like hanging out because ADHD

Do you play a musical instrument? ☐ Yes ☐ No If Yes, which
Drums

Do you play any sports? ☐ Yes ☐ No If Yes, which
Used to do soccer. Baseball/hockey at home.

What things do you most like to do?
Spend time with friends, play Lego, video games, texting

What things have caused you stress or worry over the past 12 months, including currently?
Sometimes at night, get stressed because need food, but worried won't have enough and be hungry all night. Arguments with friends at school, brother doing things not allowed. Brother and sister. Brother waking up screaming.

What are you most proud of?
Doing what parents ask; at school, won a basketball mini game; beat friend at video games for the first time

Experimenter notes

Age and gender	10; Boy
Sports	Yes (2)
Music	Yes (1)
Parent income	$50-100K
Languages	2 (French, English)
Parent education	Master's Degree (father)<br>Master's Degree (mother)
Ethnicity	Canadian (father; mother)

Referred to as SE in paper (Tested by DSL; Answers dictated to DSL)

My ADHD symptoms are:
Very hyper, don't focus as well, sometimes I get tired, sometimes I don't, have less control over my actions

Are there aspects of ADHD that you like? If so, what are they?
I like that I think outside the box a lot; I get hyperfocused. I come up with answers that others don't. It makes me more hyper than other kids my age. I get along with younger kids – in preschool and kindergarten.

What aspects of ADHD bother you the most?
The lack of patience; can't control my emotions. If my brothers are being super annoying, it's harder to walk away. I try to not hurt them, but it's not to (sic) …

Do you play a musical instrument? ☐ Yes ☐ No If Yes, which

No instrument, but I take voice lessons

Do you play any sports? ☐ Yes ☐ No If Yes, which
Gymnastics, jogging, hockey, dance, rifle

What things do you most like to do?
Sing, listen to music, family time, hanging out with my friends, doing puzzles (gets me away from the boys)

What things have caused you stress or worry over the past 12 months, including currently?
Letting people down (my director at The Lion King & my vocal teacher); not doing my best. My brother, M (7yo; 332228) wants me to play all the time but I want some alone time. I want to play cause he's away all day but it ends up having an effect on me cause I'm letting him down; not finishing my school work on time.

What are you most proud of?
My improving in math in school. I've grown closer to my brothers in the past 3 years; the play – it goes both ways. I'm nervous about and I'm proud of it. Just having an awesome family and proud of my accomplishments in gymnastics (been doing it for 3 years). Proud of helping out in my church and helping out with the younger kids. Proud of my relationship with God and how its growing more and more.

Experimenter notes

Age and gender	13; Girl
Sports	Yes (5)
Music	Yes (Voice)
Parent income	$50-100K
Languages	2 (French, English)
Parent education	Master's Degree (father); Master's Degree (mother)
Ethnicity	Canadian (father; mother)

Referred to as SF in paper (Tested by DSL; Answers written by child)

My ADHD symptoms are:
Hipper (sic). Can't foccuse (sic)

Are there aspects of ADHD that you like? If so, what are they?
When hipper (sic), creative, flexible, athletic

What aspects of ADHD bother you the most?
Can't foccuse (sic)

Do you play a musical instrument? ☐ Yes ☐ No If Yes, which

Do you play any sports? □ Yes □ No If Yes, which
Soccer, hiphop, gymnastics

What things do you most like to do?
Soccer, watch YouTube, gymnastics

What things have caused you stress or worry over the past 12 months, including currently?
When my mum and dad fight

What are you most proud of?
My soccer skills

Experimenter notes

Age and gender	9; Girl
Sports	Yes (2)
Music	Yes (1)
Parent income	$20-50K
Languages	1 (English)
Parent education	2-Year College - Degree (mother)<br>2-Year College - Degree (father)
Note	Child is adopted
Ethnicity	Unknown

Referred to as SG in paper (Tested by DSL; Answers dictated to DSL)

My ADHD symptoms are:
Time wasting and more energy. Feel like I'm being noisy at home but not in school.

Are there aspects of ADHD that you like? If so, what are they?
It makes me energetic and makes me better at baseball and sports and I can cheer my teammates on it every single pitch and it's because of my ADHD.

What aspects of ADHD bother you the most?
Very hard to focus when I'm working; usually I'm thinking of one thing and then I can't stop thinking about, usually random things.

Do you play a musical instrument? □ Yes □ No If Yes, which
Drums, piano, the bongos

Do you play any sports? □ Yes □ No If Yes, which
Soccer, baseball, swimming, California kickball, basketball, parkour

What things do you most like to do?

Baseball and playing with Pokemon toys.

What things have caused you stress or worry over the past 12 months, including currently?
When I have homework; grandpa was sick and has gone to the hospital 5 times and we didn't know if he would survive but somehow he did and he might go back again. Grandma died in 2014 -- I was thinking of it. School.

What are you most proud of?
Playing the drum. I've been playing it for 4 years; played it in front of my whole school 4 times.

Experimenter notes

Age and gender	9; Boy
Sports	Yes (6)
Music	Yes (3)
Parent income	>$100K
Languages	1 (English)
Parent education	MD (mother)<br>Bachelor's Degree (father)
Ethnicity	Western European (mother; father)

Referred to as SH in paper (Tested by KB; Answers mostly written by child; Some answers dictated to KB)

My ADHD symptoms are:
I don't know

Are there aspects of ADHD that you like? If so, what are they?
I don't know (dictated to KB)

What aspects of ADHD bother you the most?
I don't know (dictated to KB)

Do you play a musical instrument? ☐ <u>Yes</u> ☐ No If Yes, which
Drums
Do you play any sports? ☐ Yes ☐ No If Yes, which
Baseball, hockey, basketball, golf

What things do you most like to do?
Video games

What things have caused you stress or worry over the past 12 months, including currently?
School

What are you most proud of?
My city in Minecraft

Experimenter notes

Age and gender	13; Boy
Sports	Yes (4)
Music	Yes (1)
Parent income	>$100K
Languages	2 (French, English)
Parent education	Bachelor's Degree (mother)<br>Bachelor's Degree (father)
Ethnicity	Western and Eastern European (mother); Western European and Canadian (father)

Referred to as SI in paper (Tested by KB; Answers dictated to KB)

My ADHD symptoms are:
Lots of energy; make me have better listening (sic)

Are there aspects of ADHD that you like? If so, what are they?
I don't know if it's ADHD but I never want to go to sleep

What aspects of ADHD bother you the most?
Feeling stressed about doing so much stuff (work, homework)

Do you play a musical instrument? ☐ Yes <u>☐ No</u> If Yes, which

Do you play any sports? <u>☐ Yes</u> ☐ No If Yes, which
Baseball (favourite), tennis, soccer

What things do you most like to do?
I don't really remember. I really like TV and games, and video games.

What things have caused you stress or worry over the past 12 months, including currently?
Homework. People excluding me from games.

What are you most proud of?
After doing all my homework. Good hits at baseball.

Experimenter notes

Age and gender	8; Boy
Sports	Yes (3)
Music	No
Parent income	>$100K

Languages	1 (English)
Parent education	Master's Degree (mother, biological)<br>Bachelor's Degree (mother, not biological)
Ethnicity	East Asian (mother; father)

Referred to as SJ in paper (Tested by DSL; Answers partially written by child and partially dictated to DSL)

My ADHD symptoms are:
Can't stay focused. Hyper. I like math, but don't like Kumon. Have soccer – go for a very long time and stuff before then. Don't want to do it, just want to go to bed, Have a bad attitude. Dad says it and sometimes mum as well. (Dictated to DSL)

Are there aspects of ADHD that you like? If so, what are they?
Not really. (Dictated to DSL)

What aspects of ADHD bother you the most?
That I can't stay focused on my work for long periods of time. (Written by child)

Do you play a musical instrument? ☐ Yes ☐ No If Yes, which
Piano and percussion

Do you play any sports? ☐ Yes ☐ No If Yes, which
Soccer, volleyball, basketball

What things do you most like to do?
I like to play soccer and video games. I also like hanging out with my friends. (Written by child)

What things have caused you stress or worry over the past 12 months, including currently?
I have found my parents bugging me about Kumon and *unintelligible writing* constantly stresses me out. (Written by child)

What are you most proud of?
I am proud that I play Division 1 soccer.

Experimenter notes

Age and gender	14; Boy
Sports	Yes (3)
Music	Yes (2)
Parent income	>$100K
Languages	1 (English)
Parent education	Bachelor's Degree (mother)<br>> 2-Year College, but no 4-Year Degree (father)
Ethnicity	South Asian (mother; father)

Referred to as SK in paper (Tested by DSL; Answers written by child)

My ADHD symptoms are:
That I can consen (sic) trat (sic) better.
Note: The child wrote "can". It's not a data-entry error.

Are there aspects of ADHD that you like? If so, what are they?
That it makes me creative, day dreaming, consentrating (sic)

What aspects of ADHD bother you the most?
(No answer)

Do you play a musical instrument? □ Yes □ No If Yes, which
(No answer)

Note: Stepmother implied piano

Do you play any sports? □ Yes □ No If Yes, which
Soccer, snowbord (sic)

What things do you most like to do?
Play video games, snow bort (sic), bounce on a trampoline, play with Rocky.
Note: Rocky is the dog

What things have caused you stress or worry over the past 12 months, including currently?
My dog staying home

What are you most proud of?
Having a dog.

Experimenter notes

Age and gender	12; Boy
Sports	Yes (2)
Music	Yes (1)
Parent income	>$100K
Languages	3 (French, Spanish, English)
Parent education	Bachelor's Degree (mother)<br>> 2-Year College, but no 4-Year Degree (father)
Ethnicity	Latin (mother; father)

Referred to as SL in paper (Tested by DSL; Answers written by child)

My ADHD symptoms are:

Not being able to focus. Being impulsive and not being able to sit still.

Are there aspects of ADHD that you like? If so, what are they?
My hyper focus and I have a pretty good memory.

What aspects of ADHD bother you the most?
Not sitting still. It is annoying to me as well as others,

Do you play a musical instrument? ☐ Yes <u>☐ No</u> If Yes, which

Do you play any sports? <u>☐ Yes</u> ☐ No If Yes, which
Soccer

What things do you most like to do?
Swim. Climb trees. Play video games and listen (sic) to music, also bicking (sic).

What things have caused you stress or worry over the past 12 months, including currently?
Losing my keys. Getting homework done. Doing my spoken word in front of the team.

What are you most proud of?
My ability to do math. Helping out around the house and with A.
Note: A is child’s 18-month-old sister. Name redacted.

Experimenter notes

Age and gender	13; Boy
Sports	Yes (3)
Music	No
Parent income	>$100K
Languages	1 (English)
Parent education	Bachelor’s Degree (father)<br>High School (mother)<br>2-Year College – No Degree (stepmother and stepfather)
Ethnicity	Western European (father; mother)
